# Supplementary material for: Development of human hepatocellular carcinoma in X-linked severe combined immunodeficient pigs: An orthotopic xenograft model
Source: PLoS One. 2021 Mar 22;16(3):e0248352. doi: 10.1371/journal.pone.0248352 (PMC7984615; doi:10.1371/journal.pone.0248352)
Supplement: S1 Table — (DOCX) [file pone.0248352.s001.docx]

| Pig No. | Age  (Day) | Days after implantation | HepG2 cells implantation | Body weight measurement | Blood sampling | Ultrasonography | CTAP/CTHA | Necropsy |
| --- | --- | --- | --- | --- | --- | --- | --- | --- |
| 1 | 48 | 0 | + | + | – | + | – | – |
|  | 55 | 7 | – | + | – | + | – | – |
|  | 62 | 14 | – | + | – | + | – | – |
|  | 69 | 21 | – | + | – | + | – | – |
|  | 76 | 28 | – | + | – | + | – | – |
|  | 83 | 35 | – | + | – | – | – | + |
| 2 | 48 | 0 | + | + | – | + | – | – |
|  | 55 | 7 | – | + | – | + | – | – |
|  | 62 | 14 | – | + | – | + | – | – |
|  | 69 | 21 | – | + | – | + | – | – |
|  | 76 | 28 | – | + | – | + | – | – |
|  | 83 | 35 | – | + | – | – | – | + |
| 3 | 44 | 0 | + | + | – | + | – | – |
|  | 51 | 7 | – | + | – | + | – | – |
|  | 58 | 14 | – | + | – | + | – | – |
|  | 65 | 21 | – | + | – | + | – | – |
|  | 72 | 28 | – | + | – | + | – | – |
|  | 79 | 35 | – | + | – | + | – | – |
|  | 86 | 42 | – | + | – | + | + | – |
|  | 88 | 44 | – | – | – | + | – | + |
| 4 | 44 | 0 | + | + | – | + | – | – |
|  | 51 | 7 | – | + | – | + | – | – |
|  | 58 | 14 | – | + | – | + | – | – |
|  | 65 | 21 | – | + | – | + | – | – |
|  | 72 | 28 | – | + | – | + | – | – |
|  | 79 | 35 | – | + | – | + | – | – |
|  | 86 | 42 | – | + | – | + | + | – |
|  | 88 | 44 | – | – | – | + | – | + |
| 5 | 45 | 0 | + | + | + | + | – | – |
|  | 54 | 9 | – | + | + | + | – | – |
|  | 61 | 16 | – | + | + | + | – | – |
|  | 68 | 23 | – | + | + | + | – | – |
|  | 75 | 30 | – | + | – | + | + | – |
|  | 77 | 32 | – | + | + | – | – | + |
| 6 | 45 | 0 | + | + | + | + | – | – |
|  | 54 | 9 | – | + | + | + | – | – |
|  | 61 | 16 | – | + | + | + | – | – |
|  | 68 | 23 | – | + | + | + | – | – |
|  | 75 | 30 | – | + | – | + | + | – |
|  | 77 | 32 | – | + | + | – | – | + |
| 7 | 49 | 0 | + | + | – | + | – | – |
|  | 56 | 7 | – | + | + | + | – | – |
|  | 63 | 14 | – | + | + | + | – | – |
|  | 70 | 21 | – | + | + | + | – | – |
|  | 75 | 26 | – | – | – | – | – | + |
| 8 | 49 | 0 | + | + | – | + | – | – |
|  | 56 | 7 | – | + | + | + | – | – |
|  | 63 | 14 | – | + | + | + | – | – |
|  | 70 | 21 | – | + | + | + | – | – |
|  | 78 | 29 | – | + | + | + | – | – |
|  | 82 | 33 | – | + | – | + | + | – |
|  | 84 | 35 | – | + | + | + | – | + |
