# Supplementary figures and images for: Development of human hepatocellular carcinoma in X-linked severe combined immunodeficient pigs: An orthotopic xenograft model
Source: PLoS One. 2021 Mar 22;16(3):e0248352. doi: 10.1371/journal.pone.0248352 (PMC7984615; doi:10.1371/journal.pone.0248352)

## Slide 1
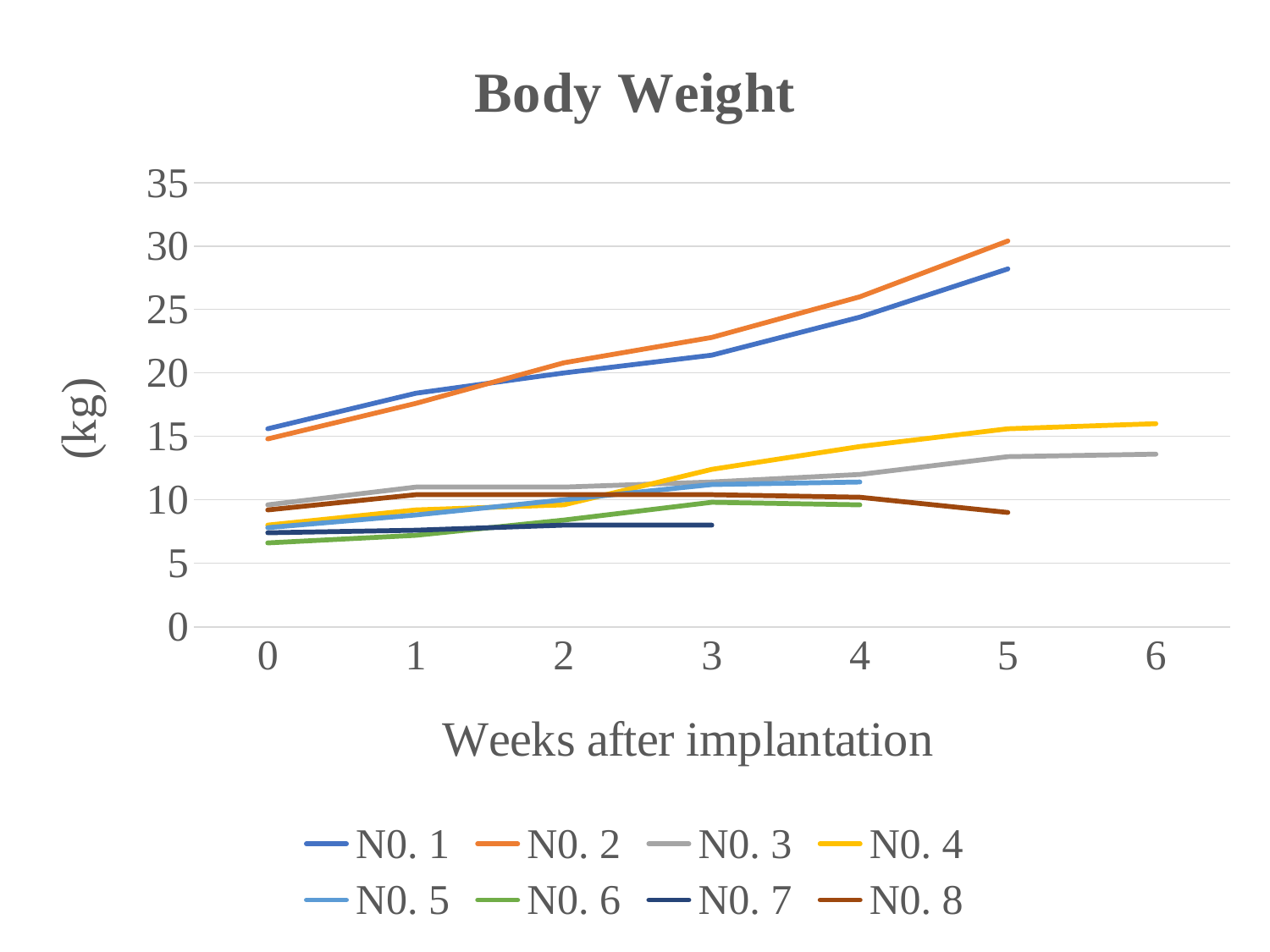

### Chart: Body Weight
| Category | N0. 1 | N0. 2 | N0. 3 | N0. 4 | N0. 5 | N0. 6 | N0. 7 | N0. 8 |
|---|---|---|---|---|---|---|---|---|
| 0 | 15.6 | 14.8 | 9.6 | 8.0 | 7.8 | 6.6 | 7.4 | 9.2 |
| 1 | 18.4 | 17.6 | 11.0 | 9.2 | 8.8 | 7.2 | 7.6 | 10.4 |
| 2 | 20.0 | 20.8 | 11.0 | 9.6 | 10.0 | 8.4 | 8.0 | 10.4 |
| 3 | 21.4 | 22.8 | 11.4 | 12.4 | 11.2 | 9.8 | 8.0 | 10.4 |
| 4 | 24.4 | 26.0 | 12.0 | 14.2 | 11.4 | 9.6 | None | 10.2 |
| 5 | 28.2 | 30.4 | 13.4 | 15.6 | None | None | None | 9.0 |
| 6 | None | None | 13.6 | 16.0 | None | None | None | None |

Supplement: S1 Fig — (PPTX) [file pone.0248352.s002.pptx]
