# Supplementary material for: Development of human hepatocellular carcinoma in X-linked severe combined immunodeficient pigs: An orthotopic xenograft model
Source: PLoS One. 2021 Mar 22;16(3):e0248352. doi: 10.1371/journal.pone.0248352 (PMC7984615; doi:10.1371/journal.pone.0248352)
Supplement: Attachment — Submitted filename: Response_to__reviewers.docx [file pone.0248352.s007.docx]

February 14, 2021

Gianfranco D. Alpini
Academic Editor

*PLOS ONE*

Dear Editor:

I wish to re-submit the revised version of our article for publication in *PLOS ONE*, titled “**Development of human hepatocellular carcinoma in X-linked severe combined immunodeficient pigs: An orthotopic xenograft model**.” The paper was co-authored by Kohei Mishima, Sachiko Matsuda, Shunichi Suzuki, Akira Onishi, Masashi Tamura, Masanori Inoue, Yuta Abe, Hiroshi Yagi, Taizo Hibi, Minoru Kitago, Masahiro Shinoda, and Yuko Kitagawa. The manuscript ID is PONE-D-20-28805.

We are thankful for the constructive comments, which have helped us to considerably improve the overall presentation of our manuscript. We have provided a point-by-point response to each reviewer comment below.

We hope that the changes incorporated into the revised manuscript satisfactorily address the reviewers’ concerns and our manuscript will now be considered suitable for publication in your journal.

We thank you for your consideration and look forward to hearing from you.

Sincerely,

Professor Osamu Itano

Department of Hepato-Biliary-Pancreas & Gastrointestinal Surgery,

International University of Health and Welfare School of Medicine,

Chiba, 286-8686, Japan.

Email: [itano@iuhw.ac.jp](mailto:itano@iuhw.ac.jp)

**Journal Requirements:**

1. Please ensure that your manuscript meets PLOS ONE's style requirements, including those for file naming.

**Response:** We have re-edited the entire manuscript to ensure that it meets your style requirements. English proofreading was performed by Editage ([www.editage.jp](http://www.editage.jp)), a company that provides professional English language editing services..

2. In your Methods section, please provide additional information on the animal research and ensure you have included details on:

(i) the frequency of animal health monitoring, including the specific criteria used to monitor animal health;

(ii) Basic housing and breeding details (pathogen-free environment, light/dark cycling, cage size).

**Response**: We have added several sentences to the Material and Methods section to address the points raised (Page 6, Line 83-87).

3. Your ethics statement should only appear in the Methods section of your manuscript. If your ethics statement is written in any section besides the Methods, please delete it from any other section.

**Response:** We have deleted the ethics statement from other sections of the manuscript apart from the methods.

**Comments from reviewers:**

Reviewer #1: In the current manuscript, Mishima et al. aimed to establish a human cell xenograft tumor model in the immunodeficient pig. The authors first tested if subcutaneous inoculation of HepG2 cells could form a tumor in the pig model. As expected, subcutaneous tumors were found in the immunodeficient pig. Thus, they did orthotopic inoculation of HepG2 cells in the pig liver. Consistently, orthotopic tumors were identified in 6 out of 8 immunodeficient pigs. Overall, the current study is simple and straightforward with significant contribution to the field. However, some concerns should be further clarified by the authors before its acceptance to be published in PLOS One.

1. The manuscript should be organized in a professionally scientific way.

**Response:** Along with English proofreading, the entire manuscript has been polished for a more scientific tone. Further, we have added radiographic data of US, CT, and macroscopic findings of SCID pigs (Fig 4, 5, and 6).

2. The HepG2 cell is a hepatoblastoma cell line.

**Response:** In addition to the hepatoblastoma cell line (PubMed: 19751877), HepG2 is also referred as HCC cell line based on the original publication (PubMed: 6248960). According to the ATCC (VA, USA, <https://www.lgcstandards-atcc.org/en/Products/All/HB-8065.aspx>), HepG2 was derived from the HCC of a 15-year-old Caucasian male.

3. Did the authors check the pig liver function by examining serum markers? It may be helpful for scientists’ reference to compare human HCC and the porcine xenograft liver cancer model.

**Response:** We performed blood sampling in four pigs (No. 5-8). AFP levels increased over time after HepG2 cell injection and this has been shown in Figure 3.

4. The xenograft model develops tumor foci in multiple locations. Is this due to micro-metastasis or just random engraftment?

**Response:** As shown in the Figure 5 (CT images) and 6 (Macroscopic findings), multinodular tumors had a certain degree of localization and appeared randomly engrafted around the local injection site. It is unknown whether tumor embolism in the portal vein or hepatic vein was due to the metastasis of the engrafted tumor or due to the HepG2 cells entering the portal vein directly at the time of HepG2 cell injection (Page 19, Line 296-300).

Reviewer #2: In this study, the authors developed a novel large-animal hepatocellular carcinoma (HCC) model, which is a xenograft model of human HCC in the X-linked interleukin-2 receptor gamma chain gene (Il2rg)-targeted severe combined immunodeficient (SCID) pigs and aimed to use this model for surgical or clinical imaging studies on HCC. HepG2 cell suspension was directly injected into the liver parenchyma of Il2rg-targeted SCID pigs via ultrasonography-guided percutaneous puncture. The authors demonstrated that the successfully generated porcine model of human HCC might enable developing better therapeutic strategies for HCC. Overall, data provided here by the authors could have interesting implications. Specific points need to be considered are listed below:

1. To better characterize the orthotopic xenograft model, the authors need to measure the serum levels of ALT, AST, etc.

**Response:** Frozen sera from four pigs were used and the timeline of AST, ALT, and AFP level monitoring is shown in Figure 3. Although no changes were observed in AST and ALT levels, an increase in AFP levels reflected tumor engraftment.

2. It’s necessary to mention the differences between pHCC and hHCC.

**Response:** In the Discussion section, we have included a sentence mentioning differences between human HCC (hHCC) and porcine HCC (pHCC) (Page 17, Line 270-271).

3. Liver weight and liver to body weight ratio should be included in Table 1.

**Response:** Unfortunately, we did not measure liver weight in this study.

4. The species of the pigs, as well as liver pathology need to be included in Table 3.

**Response:** We have added genotypic information in Table 1. Information on liver pathology has been added to Table 2.

5. The authors need to mention the gender of the pigs used for the xenograft.

**Response:** We have included gender information in Table 1. We have also included information on age, body weight, and genotype.
